# Women’s perceptions of cardiovascular risk after preeclampsia: a qualitative study

**DOI:** 10.1186/s12884-022-05179-9

**Published:** 2022-11-11

**Authors:** Lene Musfelt Nielsen, Maria Guldbrandt Hauge, Anne S. Ersbøll, Marianne Johansen, Jesper James Linde, Peter Damm, Karoline Kragelund Nielsen

**Affiliations:** 1 Department of Public Health, University of Copenhagen, Copenhagen, Denmark; 2 Department of Obstetrics, Rigshospitalet, Copenhagen University Hospital, Copenhagen, Denmark; 3 Department of Cardiology, The Heart Centre, Rigshospitalet, Copenhagen University Hospital, Copenhagen, Denmark; 4 Department of Clinical Medicine, University of Copenhagen, Copenhagen, Denmark; 5 Health Promotion Research, Steno Diabetes Center Copenhagen, Herlev, Denmark

**Keywords:** Preeclampsia, Cardiovascular disease, Qualitative research

## Abstract

**Background:**

Preeclampsia is associated with increased risk of cardiovascular disease later in life, but studies suggest that women with previous preeclampsia are not aware of this. Little is known about how these women perceive the condition and the associated long-term risks. We examined the experiences and perceptions of preeclampsia and the increased risk of cardiovascular disease (CVD) later in life among Danish women with previous preeclampsia and their attitudes towards CVD risk screening.

**Methods:**

Ten individual semi-structured interviews were conducted with women with previous preeclampsia. Data were analysed using thematic analysis.

**Results:**

We identified six themes: 1) Experiences and perceptions of being diagnosed with preeclampsia, 2) Awareness about increased risk of CVD later in life, 3) Knowledge as a precondition for action, 4) The perception of CVD risk as being modifiable, 5) Motivators for and barriers to a healthy lifestyle, and 6) Screening for CVD. Awareness of the severity of preeclampsia was limited prior to being diagnosed. Particularly among those with few or no symptoms, preeclampsia was perceived as a non-severe condition, which was further reinforced by the experience of having received very little information. Nonetheless, some women were shocked by the diagnosis and feared for the health of the offspring. Many women also experienced physical and psychological consequences of preeclampsia. Awareness of the increased risk of later CVD was lacking; yet, when informed, the women considered this to be essential knowledge to be able to act accordingly. The risk of future CVD was perceived to be partly modifiable with a healthy lifestyle, and the women expressed a need for counselling on appropriate lifestyle changes to reduce CVD risk. Other factors were also mentioned as imperative for lifestyle changes, including social support. The women were generally positive towards potential future screening for CVD because it could provide them with information about their health condition.

**Conclusions:**

After preeclampsia, women experienced a lack of knowledge on preeclampsia and the increased risk of CVD later in life. Improved information and follow-up after preeclampsia, including guidance on CVD risk reduction and support from health professionals and family, are warranted.

## Background

Preeclampsia affects 3–5% of pregnancies and is associated with maternal and perinatal morbidity and mortality [1]. Besides complications during pregnancy, preeclampsia is also associated with increased risk of later hypertension, coronary artery disease and other cardiovascular diseases (CVD) as well as increased risk of mortality [2, 3]. Therefore, The American Heart Association has listed preeclampsia as a risk factor for CVD [4], and several countries now recommend lifestyle interventions and preventive measures after preeclampsia to reduce the risk of future CVD [5]. Some studies indicate that women with previous preeclampsia are not aware of the increased risk of CVD later in life [6, 7], and others suggest that the women’s lack of awareness can be due to limited knowledge among healthcare professionals [8, 9]. However, there is very limited evidence to inform us about how women with previous preeclampsia actually perceive the condition and the associated long-term risks. Sandsæter and colleagues studied Norwegian women with a history of preeclampsia and found that their motivation for lifestyle change was linked to early information [10]. Until recently, there was no national guidelines in Denmark which recommended that women diagnosed with preeclampsia were informed about the association or about how to reduce the risk of CVD [11]. Therefore, we aimed to explore the experiences and perceptions of preeclampsia and the associated increased risk of future CVD among Danish women with previous preeclampsia and their attitudes towards CVD risk screening.

## Methods

### Study setting

This study was conducted in Copenhagen, Denmark. Denmark has a universal tax-funded healthcare system where healthcare is provided free of charge. Pregnant women are screened for preeclampsia by their general practitioner and their midwife as part of a national routine antenatal care programme. Since 2018, the Danish Society of Obstetrics and Gynaecology (DSOG) has recommended that women with previous preeclampsia are informed about the increased risk of CVD later in life. It is also recommended by DSOG that health promoting interventions are discussed when relevant, e.g. annual blood pressure measurement and advise on a life without tobacco [12]. Today, the health authorities in Denmark recommend annual blood pressure measurement [11], but until recently there was no Danish national clinical guideline regarding time interval for clinical follow-up or preventative intervention to reduce the future CVD risk for women with previous preeclampsia.

### Study design, participants, and data collection

We conducted a qualitative phenomenological study based on semi-structured interviews with women with previous preeclampsia. Participants were recruited from The Copenhagen Preeclampsia and Cardiovascular Disease Study (CPH-PRECIOUS study) [13]. This study was approved by the Danish Data Protection Agency (VD-2018–506) and the Capital Region’s Committee on Biomedical Research Ethics (H-18065695). Women who had consented to participate in a semi-structured interview were invited to participate in the current study. Potential participants were contacted by telephone, informed about the qualitative study and invited for an interview. A total of 24 potential participants were contacted, of which 10 agreed to participate. A busy schedule was the primary reason given by participants for declining the invitation. During recruitment, we sought to achieve maximum variation in terms of age, severity of preeclampsia, comorbidities and educational level [14]. All interviews were conducted by LMN from August to October 2020. LMN was at the time a final-year MSc Public Health student and had both theoretical knowledge and practical experience with conducting qualitative interviews. LMN was not involved in patient care. The participants decided the location for the interviews, which were held in participants’ homes, in offices at Rigshospitalet or at the University of Copenhagen, at participants’ workplace or via a virtual platform. Participants gave written informed consent to participate and verbal consent for the interviews to be audio recorded. The women were not compensated for their participation.

Participants were told that there were no right or wrong answers and that they could skip questions they did not wish to answer. The interview-guide consisted of both open-ended and closed questions. Some questions aimed to examine the participants’ knowledge and understandings, while others aimed to explore and examine experiences with preeclampsia and perceptions of CVD risk. Participants were asked about their knowledge and experiences with preeclampsia, their knowledge regarding the association between preeclampsia and CVD later in life, their risk perceptions, as well as their views on future CVD risk screening. The interviews lasted 45 – 90 min.

### Data analysis

All interviews were audio recorded and transcribed verbatim to ensure that the participants’ statements were maintained in the transcripts. A thematic analysis was used to examine data and themes arising from the interviews [15]. Open and axial coding were used to code the interview transcripts and organise them into categories and themes following an abductive approach [16]. LMN coded the interviews, and the following themes were thoroughly discussed with KKN and subsequently also with the rest of the author group.

## Results

The 10 women, who participated in the study, were between 38 and 53 years old and differed according to education and occupation. Information about the participants and their preeclampsia diagnosis are presented in Table 1.

**Table 1:** Participant characteristics

		**Number. of participants**
**Age at interview (years)**	38–40	1
	41–45	2
	46–50	2
	51–53	5
**Year of preeclampsia diagnosis**^**a**^	1992–1999	5
	2000–2008	6
	2009–2017	2
**Years since first preeclampsia diagnosis**	< 15	3
	≥ 15	7
**Number of pregnancies diagnosed with preeclampsia**	1	7
	> 1	3
**Parity**	1	3
	2	4
	3	2
	4 +	1
**Experienced symptoms at the time of preeclampsia diagnosis**	No	6
	Yes	4
**Perinatal loss**	Yes	2
	No	8

^a^ Does not sum up to 10 because some participants had preeclampsia several times

Six themes emerged from the data analysis: 1) Experiences and perceptions of being diagnosed with preeclampsia, 2) Awareness about increased risk of CVD later in life, 3) Knowledge as a precondition for action, 4) The perception of CVD risk as being modifiable, 5) Motivators for and barriers to a healthy lifestyle and 6) Screening for CVD. The six themes are presented in the following.

### Experiences and perceptions of being diagnosed with preeclampsia

Most women were aware of preeclampsia before they were diagnosed themselves; however, the majority were unaware of the potentially severe course of the disease during pregnancy. Some women said they were surprised to be diagnosed with preeclampsia because they did not feel any symptoms or sensations. The lack of symptoms entailed for some women that they initially did not perceive preeclampsia as a potentially severe disease when they were diagnosed. Woman 5, who did not experience any symptoms, said:

“(…) when they asked me to lay down, and I was not allowed to get up or pee or anything. I was like “Really? I’m not that sick!” – Woman 5, first diagnosed with preeclampsia 20 years ago.

Most of the women said they were not given much information about preeclampsia from the healthcare professionals when they were diagnosed, which gave them the perception that preeclampsia was not a severe condition. Nonetheless, some women reacted to the diagnosis with shock, anxiety and fear for the health and wellbeing of their unborn child. Woman 4, who had experienced symptoms, explained it like this:

“(…) I had not read those chapters in the book. I did not know what preeclampsia was. So, I thought well now you get a brain-damaged child” – Woman 4, first diagnosed with preeclampsia 21 years ago.

Thus, the lack of awareness about preeclampsia led to enhanced fears for its consequences, particularly concerning the offspring. Indeed, the women generally noted being more concerned for their unborn child than for their own health. Preeclampsia disappeared shortly after delivery, leading to the perception that preeclampsia and its associated risks were over among several participants:

“(…) I would call it symptoms in pregnancy. It was just like, as soon as I delivered my girl, it was all over. (…) I don’t see it as a chronic disease” – Woman 1, first diagnosed with preeclampsia 21 years ago.

The perception that the risks associated with preeclampsia were temporary and ceased with the delivery were reported by the women regardless of whether they had experienced any symptoms or complications during pregnancy. Woman 2 described that she had had symptoms and complications after both preeclampsia and delivery. She experienced both a depressive state and later also CVD, and said:

“And you also think it is not something that will harm you, but it actually is – which I found out afterwards, right?” – Woman 2, first diagnosed with preeclampsia 21 years ago.

Yet, as also indicated by this quote, some women discovered that the risks were not over, even though preeclampsia disappeared. Some women were still physically affected due to preeclampsia postpartum and experienced hypertension and/or liver and kidney damages long after the delivery. Others were affected psychologically; they developed depression or depression-like conditions and several women experienced the hospitalisation before and after delivery as putting a strain on them mentally. For some, preeclampsia also had implications for their experience of motherhood. Two women experienced perinatal loss due to complications during pregnancy or delivery, and others were subsequently recommended by their doctor not to become pregnant again due to the increased risk of relapse of preeclampsia.

### Awareness about increased risk of CVD later in life

Before the participation in the CPH-PRECIOUS study, only two of the women said they had been aware of the increased risk of future CVD after a pregnancy complicated by preeclampsia. One woman was informed because she subsequently developed chronic hypertension, and the other woman was told about the risk and possible risk factors for CVD after preeclampsia by the doctor at the hospital. When learning about the subsequent risk of CVD, the women experienced various reactions. Some women were not surprised about the association with future CVD because they noted it involved some of the same symptoms, e.g. high blood pressure, headache etc. Nevertheless, some women found it difficult to cope with this information, which made them feel anxious and worried about their future health. When talking about her reaction to learning about the future risk of CVD, woman 6, who had severe complications from preeclampsia, said:

“(…) [I thought the information was] terrifying. Not only did you have to go through a process with preeclampsia, and then [you find out] it could also have consequences later in life (…). I thought it was a thing like, now it is over. But that it could spread like ripples in a pond. I thought that was really frightening, really frightening.” – Woman 6, first diagnosed with preeclampsia 6 years ago.

Other women were less worried because it only was a *possibility* and not something that would develop *for certain*. A couple of the women, who had already developed CVD and had not been aware of the association before participating in the CPH-PRECIOUS study, reflected on the possibility that the CVD could be a result of the previous preeclampsia – a link they had not made previously:

“(…) I thought: oh, so maybe it is not just stress or hereditary disposition, right? My God, there is also a factor here, which I have not thought about actually!” – Woman 9, first diagnosed with preeclampsia 25 years ago.

There were also some women who already considered themselves to be at risk of CVD even prior to their pregnancy with preeclampsia. Some already had symptoms of CVD before their pregnancy and others because of known hereditary dispositions. These women did not consider their perceived CVD risk as being changed after receiving information about the association with preeclampsia, simply because they already perceived themselves as being at high risk. Woman 4, who considered herself as having a hereditary disposition, noted that she did not perceive her risk of CVD as being higher despite the association:

“So, I don’t want to say that it is a double risk (…) I just have a risk to take. I cannot do any more than I already do (…) I might as well park it because I cannot do anymore (…). It’s the same [risk]” – Woman 4, first diagnosed with preeclampsia 21 years ago.

While knowledge about the association between preeclampsia and CVD had not resulted in any additional preventive action for woman 4, it was considered a precondition for action by other women as described in the next theme.

### Knowledge as a precondition for action

According to the women, the most important reason to be informed about the association between preeclampsia and CVD was the possibility to act on the risk themselves. The women requested knowledge about the association and preventive strategies, so they themselves could make an informed decision as to whether and how to act upon the given information. When asked about why she wanted information, woman 10 replied:

“[To know about] what you can do yourself, and – if you are in an at-risk group – what you can do to avoid it. Should you take fish oil capsules? Or should you run up the stairs? Or should you stop drinking ten Pepsi Max? Or whatever. It could be some health, yes, some health information on what it requires if you want to prevent it. Like, I might be doing things I don’t have to, and it might even be better if I do yoga, run on stairs or whatever, right?” – Woman 10, first diagnosed with preeclampsia 13 years ago.

The women also made suggestions on how information about the association between preeclampsia and CVD could be provided. A few of the women thought that the healthcare professionals should just straight-up tell women with preeclampsia about facts and not be afraid to tell them about the association simply because it is important to be aware of:

“They [healthcare professionals] should not be afraid to alarm the patients. I really think the important thing is to get everything on the table. If you as a patient don’t know about this association, you might not be aware of just how important it is to mind your medication and to measure your blood pressure regularly – you may come to take it too lightly” – Woman 7, first diagnosed with preeclampsia 20 years ago.

The women had several specific suggestions on how to get information in form of facts, numbers and statistics. For instance, information on prevalence, general and personal risk, and how much the risk can be changed through healthy living. Many women also wanted more information from the hospital or doctor, especially regarding what they personally could do to prevent their own risk. Some women suggested disseminating such information to raise awareness through tv-shows, teaching, lectures or in community groups for new mothers.

### The perception of CVD risk as being modifiable

While knowledge about the risk of subsequent CVD was considered a prerequisite for action, it was not in itself sufficient to ensure action. According to the women, other factors were of importance as well, e.g. skills and knowledge on how to make changes. Also, the perception of being able to actually control or influence the risk of CVD was mentioned. Further, while family inheritance was mentioned as a CVD risk factor the women could not influence themselves, the majority of women considered themselves as having some ability to control their health and thereby their risk of CVD:

“(…) okay, so it is a risk, but it is not certain that this [CVD] will happen. And secondly, it is something that we can handle. Like for example you can make sure that your lifestyle is such that you reduce the risk. Blood pressure medicine can lower your blood pressure. So, you can do all sorts of things (…)” – Woman 8, first diagnosed with preeclampsia 12 years ago.

When talking about what could be done to control the risk of CVD, the women mostly mentioned risk factors such as a diet, exercise and stress. The women considered these factors as something they themselves could influence and thereby prevent the development of CVD. However, the perception of being able to influence or control the risk of CVD itself also meant that several of the women perceived it to be one’s own responsibility:

“You can make a difference, but you cannot make the whole difference (…) So I think that sometimes we are told that it is our own responsibility (…) And of course you have a responsibility, I believe that you do (…) – Woman 3, first diagnosed with preeclampsia 28 years ago.

As reflected in the quote from woman 3, the perception of CVD development as the individual’s own responsibility was also a belief that was enforced by statements from the surroundings, including healthcare professionals.

### Motivators for and barriers to a healthy lifestyle

Most of the interviewed women believed that they currently had a healthy lifestyle. Nonetheless, a few said they still wanted to make some lifestyle changes. The women described different kinds of reasons for wanting to obtain or maintain a healthy lifestyle, including wanting to lose weight, vanity, staying healthy and live a long life for the sake of their family, having a good physical and mental health, and wanting to reduce sickness and risks for disease, and in case illness could not be avoided, to improve one’s chances of managing it. When talking about what motivated her to live healthy, woman 1 said:

“To make myself stronger both mentally and physically, and to handle that kind of disease, so (…) if I get the disease (…) I will know that I have a strong body, which can receive and fight it” – Woman 1, first diagnosed with preeclampsia 21 years ago.

Moreover, the social aspects of health and healthy living were highlighted by one woman. Woman 8 described the ‘social motivation’ she experienced when it came to healthy living:

“I also think that there is something social about living healthy. The social motivation is indeed high. Well, among my friends and family (…) living healthy is something you do. It is a thing that just belong to it really (…)” – Woman 8, first diagnosed with preeclampsia 12 years ago.

The participants also mentioned barriers to a healthy lifestyle. These included family and own (old) eating habits, lack of time, sickness, bad weather, temptations, and lack of support from family and doctor. Woman 2 had gained a substantial amount of weight and had been diagnosed with stroke. In the interview she described how she had difficulties being physically active and needed to lose weight, and how she had tried to ask her doctor for help:

“(…) I have tried to talk to him [the doctor] (…) about my diet, where I asked him, if he could help me to get to one of those people who can help you with your diet [a dietician] (…). But no, he could not help me (…). Then I said, ‘well why not? It is a big problem (…) I should not put on too much weight from now on, right?’ But no, he could not help with that (…) it was not a part of what he should (…) talk to his patients about.” – Woman 2, first diagnosed with preeclampsia 21 years ago.

While lack of support from healthcare professionals were described by some women, those who wanted to make lifestyle changes expressed an interest in guidance from healthcare professionals.

### Screening for CVD

The women were asked about their opinions and perceptions of attending a CVD risk screening programme if one was to be implemented. All the women expressed a positive attitude towards a potential screening programme specifically for CVD risk following a preeclampsia diagnosis, and most said they would participate in such a screening programme if offered. The main reasons given for the willingness to participate in a CVD risk screening programme were to obtain knowledge and to prevent disease. Woman 8 said:

“(…) I think there is no reason not to accept such an offer, if it somehow can give me some knowledge that I have to consider or so that I then can take some preventive measures.” – Woman 8, first diagnosed with preeclampsia 12 years ago.

Thus, according to the women, screening was an opportunity to acquire knowledge on their health. Knowledge which would enable them to form an informed decision and potentially take precautionary action. However, some women also noted that there could be negative aspects of screening programmes as well. Talking about screening programmes in general, woman 7 for instance noted:

“Screening is a great tool that makes it possible to catch serious illness early, but of course the drawback is that you may alarm the patient. It is unpleasant, but it cannot be helped. This makes communication extra important” – Woman 7, first diagnosed with preeclampsia 20 years ago.

Thus, while the women acknowledged that e.g. receiving a false positive or negative result could cause concerns and worries, for many participants it did not change their overall positive view of screening.

## Discussion

This study explored the experiences and perceptions of preeclampsia and the increased risk of future CVD among Danish women diagnosed with preeclampsia during 1992–2017 and their attitudes towards CVD risk screening. Our study showed that women with previous preeclampsia had limited awareness about preeclampsia before they were diagnosed, and they did not experience receiving much information about the diagnosis, which entailed a perception that preeclampsia was not a severe condition. The women in our study had experienced physical and psychological consequences of preeclampsia, including damage to kidney and liver, depression, and perinatal loss. Our study also suggests a low level of knowledge of the association between preeclampsia and risk of future CVD before women participated in the CPH-PRECIOUS study. Information about the association caused different reactions; some were not surprised, whereas others became worried about future health. Some of the women, who already had developed CVD, reflected on whether preeclampsia could be a cause to their CVD now they had become aware of the association. For most of the participants information on the association between preeclampsia and future CVD was essential to act on the CVD risk themselves, though it was not sufficient to initiate action. Factors, such as guidance to healthy lifestyle and support from family and healthcare professionals, were also mentioned as necessary for behaviour change or maintaining healthy behaviours. Perceptions of ‘being in control’ to prevent CVD were highlighted and some noted feeling a responsibility for being healthy. Many women perceived their lifestyle as being healthy, but some wanted professional help to make lifestyle changes to reduce CVD risk. A motivator for a healthy lifestyle was to stay healthy for both their family and their own sake. Barriers included lack of support from family and doctors. The women were positive about participation in a potential screening programme for CVD after preeclampsia, mainly because it would provide knowledge on their health condition.

Existing evidence on the topic is scarce. However, similar results have been documented in a couple of previous studies. The perception that preeclampsia is not a severe condition was also found in a focus-group study among women from the United States, who reported experiencing that their doctors did not explain the diagnosis [7]. A questionnaire study, also among women in the United States, likewise found that women were unaware of preeclampsia before they were diagnosed with it themselves [17]. We further show that some women reacted with surprise, shock and anxiety after being diagnosed. The feeling of surprise and being unprepared of the diagnosis has also been noted in an Australian study where women were interviewed 10–12 months postpartum [18], and similar reactions have been documented among women with other diseases in pregnancy, e.g. gestational diabetes mellitus [19]. Among the women in our study, preeclampsia had serious reproductive consequences for the women: some decided not to have more children and two women experienced perinatal loss. Although perinatal mortality is estimated to occur in only 1–2% of severe preeclampsia cases [20], our findings highlight that, among those affected, preeclampsia is a serious disease which can have substantial impact on both women and their children in the short- and long-term. In other studies, the decision not to have more children was due to the women’s anxiety for relapse of preeclampsia which was found in a Norwegian focus-group study [10], or due to a course of severe sickness with preeclampsia which was documented in a study among British women, who attended a postnatal clinic three months after delivery [6]. In our study, a few women were recommended by their doctors not to have more children due to the severity of their preeclampsia. Thus, it was not only the women’s own experiences that influenced the decision of not having more children, which emphasises the importance of the doctor’s recommendations on such a decision.

Only few women in our study were aware of the association between preeclampsia and increased risk of CVD later in life. Until recently there were no national, official recommendations for follow-up or information about increased risk of CVD for women with prior preeclampsia in Denmark. Therefore, it is not surprising that the women in our study were not aware of the association or how to reduce the risk of CVD. Other studies have found similar low levels of awareness of this association [6, 7, 21, 22]. Reactions to receiving information about the association between preeclampsia and later CVD ranges from getting angry and scared [7] to not reporting any concern [6]. We found that being informed about the association affected some women’s risk perception, but not all; mainly because some women already had CVD or perceived themselves at being at high risk of CVD due to hereditary dispositions or because they perceived themselves as already having a healthy lifestyle. This may in part be due to the fact that for many of the women in our study more than 15 years had passed since their first preeclampsia diagnosis. Our study confirms the finding also noted in other studies that women with previous preeclampsia wish for more information about the association between preeclampsia and CVD [7, 10, 22].

Empowerment and the ability to act on the increased risk were reasons why many of the women in our study found it important to be told about the association and how to manage it. Knowledge of the association has previously been found to increase motivation for lifestyle changes in other studies [7]. Similar to our findings, a Dutch study showed motivators for a healthy lifestyle to be the prospect of having a good physical and mental wellbeing and a way to promote future health [23]. Specifically, the women in our study mentioned that having a healthy lifestyle could prepare the body for later disease and alleviate a possible course of CVD. In our study, screening was perceived to be a tool to obtain knowledge and information about own health status, and thereby a means to inform about CVD risk and prevention. Yet, in our study the women also mentioned concerns about screening. Concerns about false negative and positive results in connection with screening are known among persons with other conditions [24]. Nevertheless, women in our study said they would participate in a potential screening programme for CVD after preeclampsia because it could provide them with information that could be used to optimise their health.

Our results indicate that women with previous preeclampsia generally would like to know about the increased risk of CVD later in life and how to reduce it. Thus, there may be an unutilised potential for prevention of CVD, and official guidance, recommendations, and follow-up for this group of women might be a possible way to provide the requested information and support to women with prior preeclampsia about preventive measurements. The, until recently, lack of Danish national recommendations for these women may explain the sparse knowledge and awareness of the association between preeclampsia and CVD and how to minimise the associated CVD risk. Who and when to screen as well as whether systematic screening for CVD is effective remains unclear; however, our study emphasises that women with preeclampsia should at least be informed about the association. Yet, as this study suggests, information will not in itself be sufficient to motivate and ensure action aimed at CVD risk reduction. Information, guidance, support and tools to prevent CVD risk are needed as well. A feasible way to do this could be via national recommendations and training of relevant health professionals.

### Strengths and limitations

The study has various strengths and limitations, which should be noted. It is a strength that the women were recruited from the CPH-PRECIOUS study where the preeclampsia diagnosis was based on a validated clinical database and additionally confirmed by the participants, as this has been an issue in other studies [7, 22]. Moreover, the participants’ different characteristics according to age, education, severeness of disease, experiences of complications, and the time since preeclampsia, which was between 3–28 years ago, widens the perspective [14], and provides insights from women who experienced preeclampsia long time ago, as well as from women with recent experiences with a preeclampsia diagnosis. Nevertheless, the participants all lived in the Copenhagen area and had agreed to participate in the CPH-PRECIOUS study. Moreover, some potential participants declined the invitation, e.g., due to a busy schedule. Therefore, it is possible that women in this study represents a certain subgroup among women with previous preeclampsia and that the participants’ knowledge and interest in the subject, for instance, differ from that of other women with previous preeclampsia, who live in other parts of the country or did not wish to participate in this research. In previous studies, interviews were held between six months and five years after the diagnosis of preeclampsia [6, 7, 10, 22]. However, for some participants in our study, it had been a long time since they were diagnosed with preeclampsia, which may have made it difficult for them to remember their experiences and perceptions of preeclampsia at the time of diagnosis. It should be noted that the findings reflect their perceptions of preeclampsia at the time of the interviews rather than at the time of diagnosis. It is also important to keep in mind that the knowledge about the association between preeclampsia and CVD has changed since most of the women in our study had preeclampsia.

Thus, the diversity in characteristics of the participants and the fact that their experiences occurred in a specific context means that our findings are not necessarily transferrable to other contexts. Finally, in qualitative research it is important that the researchers acknowledge their own role in the production of the research as they are part of the world they study and not an objective, separate tool. Our backgrounds in medicine and public health may have led us to pay more attention to certain aspects or patterns in the data, e.g. prevention of disease, compared to researchers with other backgrounds. We tried to minimise individual bias via involvement of and discussions amongst several researchers in the analysis phase.

## Conclusions

Our qualitative study on the experiences and perceptions of preeclampsia and risk of CVD later in life among Danish women with previous preeclampsia and their attitudes towards CVD risk screening show that while some women knew about preeclampsia before they were diagnosed, others were shocked and feared for their unborn child’s life after being diagnosed. Many experienced a lack of information about preeclampsia, and not all perceived preeclampsia as a severe disease; yet some experienced physical, psychological, and personal consequences of preeclampsia. Only few women were aware of the association between preeclampsia and the future risk of CVD, and only rarely had the women been informed about the association with future CVD and how to prevent it. The women had different views and perceptions of the increased risk of CVD later in life, which should be considered in future CVD risk reduction intervention programmes. All participants agreed that more information about the association and help to prevent CVD and obtain a healthy lifestyle are needed. The women believed that they themselves could prevent CVD, at least partially; however, to ensure support for healthy lifestyle and timely detection of CVD, follow-up and screening for CVD risk factors after preeclampsia should be considered.

## Data Availability

The data generated and analysed in this study are not publicly available as the participants in the study did not consent to having the full transcripts of the interviews made publicly available. Making these available would hamper participant privacy.

## Abbreviation

CVD: Cardiovascular disease
CPH-PRECIOUS study: The Copenhagen Preeclampsia and Cardiovascular Disease Study
DSOG: Danish Society of Obstetrics and Gynaecology
